# Supplementary material for: Targeting Transcriptional Regulators Affecting Acarbose Biosynthesis in Actinoplanes sp. SE50/110 Using CRISPRi Silencing
Source: Microorganisms. 2024 Dec 24;13(1):1. doi: 10.3390/microorganisms13010001 (PMC11767292; doi:10.3390/microorganisms13010001)
Supplement: Supplementary file 1 [file microorganisms-13-00001-s001.zip › microorganisms-3341722-supplementary.pdf]

Supplementary materials: Targeting transcriptional regulators affecting acarbose biosynthesis in *Actinoplanes* sp. SE50/110 by CRISPRi silencing

Saskia Dymek [1], Lucas Jacob [1], Alfred Pühler [2] and Jörn Kalinowski [1,*]

[1] Microbial Genomics and Biotechnology, Center for Biotechnology, Bielefeld University, 33615 Bielefeld, Germany; sdymek@cebitec.uni-bielefeld.de (S.D.); ljacob@cebitec.uni-bielefeld.de (L.J.)
[2] Senior Research Group in Genome Research of Industrial Microorganisms, Center for Biotechnology, Bielefeld University, 33615 Bielefeld, Germany; puehler@cebitec.uni-bielefeld.de
* Correspondence: joern@cebitec.uni-bielefeld.de

Table S1. Bacterial strains used in this study.

Strain	Genotype	Reference
	E. coli strains	
E. coli DH5α	F^- φ80*lac*ZΔM15 Δ(*lac*ZYA-argF)U169 *rec*A1 *end*A1 *hsd*R17(r_K^-, m_K^+) *pho*A *sup*E44 λ^-*thi*-1 *gyr*A96 *rel*A1	[42]
E. coli ET12567/pUZ8002	*dam-13*::Tn9 *dcm-6 hsdM* Cmr	[43]
	Actinoplanes strains	
Actinoplanes sp. SE50/110	Wildtype (WT) ATCC 31044	[45]
pCR_dCas9	WT carrying the pCRISPomyces2i plasmid	This work
pCR_dCas9_*acbB*^{SI}	WT carrying the pCRISPomyces2i plasmid with *acbB* sgRNA	This work
pCR_dCas9_*acbV*^{SI}	WT carrying the pCRISPomyces2i plasmid with *acbV* sgRNA	This work
pCR_dCas9_*cadC*^{SI}	WT carrying the pCRISPomyces2i plasmid with *cadC* sgRNA	This work
pCR_dCas9_*acbB*^{CU}	pCR_dCas9_*acbB*^{SI} cured from the respective plasmid	This work
pCR_dCas9_*acbV*^{CU}	pCR_dCas9_*acbV*^{SI} cured from the respective plasmid	This work
pCR_dCas9_*cadC*^{CU}	pCR_dCas9_*cadC*^{SI} cured from the respective plasmid	This work
pS_dCas9	WT carrying the pSETT4i plasmid	This work
pS_dCas9_*acbB*	WT carrying the pSETT4i plasmid with *acbB* sgRNA	This work
pS_dCas9_*acbV*	WT carrying the pSETT4i plasmid with *acbV* sgRNA	This work
pS_dCas9_*cadC*	WT carrying the pSETT4i plasmid with *cadC* sgRNA	This work
pS_dCas9_ACSP50_*0038*	WT carrying the pSETT4i plasmid with ACSP50_*0038* sgRNA	This work
pS_dCas9_ACSP50_*0424*	WT carrying the pSETT4i plasmid with ACSP50_*0424* sgRNA	This work
pS_dCas9_ACSP50_*0500*	WT carrying the pSETT4i plasmid with ACSP50_*0500* sgRNA	This work
pS_dCas9_ACSP50_*0946*	WT carrying the pSETT4i plasmid with ACSP50_*0946* sgRNA	This work
pS_dCas9_ACSP50_*0976*	WT carrying the pSETT4i plasmid with ACSP50_*0976* sgRNA	This work
pS_dCas9_ACSP50_*0989*	WT carrying the pSETT4i plasmid with ACSP50_*0989* sgRNA	This work
pS_dCas9_ACSP50_*1196*	WT carrying the pSETT4i plasmid with ACSP50_*1196* sgRNA	This work
pS_dCas9_ACSP50_*1252*	WT carrying the pSETT4i plasmid with ACSP50_*1252* sgRNA	This work
pS_dCas9_ACSP50_*1572*	WT carrying the pSETT4i plasmid with ACSP50_*1572* sgRNA	This work
pS_dCas9_ACSP50_*1607*	WT carrying the pSETT4i plasmid with ACSP50_*1607* sgRNA	This work
pS_dCas9_ACSP50_*1631*	WT carrying the pSETT4i plasmid with ACSP50_*1631* sgRNA	This work
pS_dCas9_ACSP50_*1755*	WT carrying the pSETT4i plasmid with ACSP50_*1755* sgRNA	This work
pS_dCas9_ACSP50_*1816*	WT carrying the pSETT4i plasmid with ACSP50_*1816* sgRNA	This work
pS_dCas9_ACSP50_*1877*	WT carrying the pSETT4i plasmid with ACSP50_*1877* sgRNA	This work
pS_dCas9_ACSP50_*2235*	WT carrying the pSETT4i plasmid with ACSP50_*2235* sgRNA	This work

pS_dCas9_ACSP50_*2344*	WT carrying the pSETT4i plasmid with ACSP50_*2344* sgRNA	This work
pS_dCas9_ACSP50_*2411*	WT carrying the pSETT4i plasmid with ACSP50_*2411* sgRNA	This work
pS_dCas9_ACSP50_*2872*	WT carrying the pSETT4i plasmid with ACSP50_*2872* sgRNA	This work
pS_dCas9_ACSP50_*3384*	WT carrying the pSETT4i plasmid with ACSP50_*3384* sgRNA	This work
pS_dCas9_ACSP50_*3560*	WT carrying the pSETT4i plasmid with ACSP50_*3560* sgRNA	This work
pS_dCas9_ACSP50_*4202*	WT carrying the pSETT4i plasmid with ACSP50_*4202* sgRNA	This work
pS_dCas9_ACSP50_*4228*	WT carrying the pSETT4i plasmid with ACSP50_*4228* sgRNA	This work
pS_dCas9_ACSP50_*4364*	WT carrying the pSETT4i plasmid with ACSP50_*4364* sgRNA	This work
pS_dCas9_ACSP50_*4574*	WT carrying the pSETT4i plasmid with ACSP50_*4574* sgRNA	This work
pS_dCas9_ACSP50_*4697*	WT carrying the pSETT4i plasmid with ACSP50_*4697* sgRNA	This work
pS_dCas9_ACSP50_*4846*	WT carrying the pSETT4i plasmid with ACSP50_*4846* sgRNA	This work
pS_dCas9_ACSP50_*4891*	WT carrying the pSETT4i plasmid with ACSP50_*4891* sgRNA	This work
pS_dCas9_ACSP50_*5191*	WT carrying the pSETT4i plasmid with ACSP50_*5191* sgRNA	This work
pS_dCas9_ACSP50_*5298*	WT carrying the pSETT4i plasmid with ACSP50_*5298* sgRNA	This work
pS_dCas9_ACSP50_*5377*	WT carrying the pSETT4i plasmid with ACSP50_*5377* sgRNA	This work
pS_dCas9_ACSP50_*6401*	WT carrying the pSETT4i plasmid with ACSP50_*6401* sgRNA	This work
pS_dCas9_ACSP50_*6463*	WT carrying the pSETT4i plasmid with ACSP50_*6463* sgRNA	This work
pS_dCas9_ACSP50_*6528*	WT carrying the pSETT4i plasmid with ACSP50_*6528* sgRNA	This work
pS_dCas9_ACSP50_*6859*	WT carrying the pSETT4i plasmid with ACSP50_*6859* sgRNA	This work
pS_dCas9_ACSP50_*7140*	WT carrying the pSETT4i plasmid with ACSP50_*7140* sgRNA	This work
pS_dCas9_ACSP50_*7246*	WT carrying the pSETT4i plasmid with ACSP50_*7246* sgRNA	This work
pS_dCas9_ACSP50_*7269*	WT carrying the pSETT4i plasmid with ACSP50_*7269* sgRNA	This work
pS_dCas9_ACSP50_*7677*	WT carrying the pSETT4i plasmid with ACSP50_*7677* sgRNA	This work
pS_dCas9_ACSP50_*7823*	WT carrying the pSETT4i plasmid with ACSP50_*7823* sgRNA	This work
pS_dCas9_ACSP50_*7951*	WT carrying the pSETT4i plasmid with ACSP50_*7951* sgRNA	This work
pS_dCas9_ACSP50_*7958*	WT carrying the pSETT4i plasmid with ACSP50_*7958* sgRNA	This work
pS_dCas9_ACSP50_*7982*	WT carrying the pSETT4i plasmid with ACSP50_*7982* sgRNA	This work
pS_dCas9_ACSP50_*8007*	WT carrying the pSETT4i plasmid with ACSP50_*8007* sgRNA	This work
pS_dCas9_ACSP50_*8014*	WT carrying the pSETT4i plasmid with ACSP50_*8014* sgRNA	This work
pS_dCas9_ACSP50_*8048*	WT carrying the pSETT4i plasmid with ACSP50_*8048* sgRNA	This work
pS_dCas9_ACSP50_*8114*	WT carrying the pSETT4i plasmid with ACSP50_*8114* sgRNA	This work
pS_dCas9_ACSP50_*8120*	WT carrying the pSETT4i plasmid with ACSP50_*8120* sgRNA	This work
pS_dCas9_ACSP50_*8173*	WT carrying the pSETT4i plasmid with ACSP50_*8173* sgRNA	This work
pS_dCas9_ACSP50_*8200*	WT carrying the pSETT4i plasmid with ACSP50_*8200* sgRNA	This work
pS_dCas9_ACSP50_*8287*	WT carrying the pSETT4i plasmid with ACSP50_*8287* sgRNA	This work

Table S2. Oligonucleotides used in this study.

Name	sequence (5′ → 3′)
H840A_fw	cgactacgacgtcgacGCCatcgtgccgcagtccttcc
H840A_rv	ggactgcggcacgatGGCgtcgacgtcgtagtcgctcag
D10A_fw	gtacagcatcggcctgGCCatcggcaccaacagcgtg
D10A_rv	gctgttggtgccgatGGCcaggccgatgctgtacttct
ApmR_split_fw	gtgcaatgtcgtgcaatacg
pSET_bb_rv	ccgatgctgtacttcttgtccatgtaccatcggaatacctccgt
pSET_dCAS9_fw	gcaacggaggtattccgatggtacatggacaagaagtacagcatcgg
ApmR_split_rv	cgtattgcacgacattgcac
L438-BbsBsaxc-part1-fwd	cgagtatctgaaaggggatacgcagagacctcagccgctacagggcgcgtc
Bbs_zu_Bsa_tracr_rv	gctatttctagctctaaaacagagaccaccggtggaaagcgggc
Bbs_zu_Bsa_tracr_fw	gcccgctttccaccggtggtctctgttttagagctagaaatagc
L437-BbsBsaxc-BB-rev	gcgtatcccctttcagatactcg

qRT_*cadC*_fw	ctattgaacgcgccgacgc
qRT_*cadC*_rv	cttctcgcgctccagcagac
qRT_*acbB*_fw	gtcgacaaactgggttacgg
qRT_*acbB*_rv	gtccagtagcacctgagtg
qRT_*acbV*_fw	gcttccacggcaagacgatg
qRT_*acbV*_rv	gcgctcacgttgggtttctc

Table S3. Annealing oligonucleotides for construction of sgRNA. Overlaps are shown in capital letters.

Name	sequence (5′ → 3′)
*cadC*_sp1_fw	ACGCatgcaggagaatcgatgcag
*cadC*_sp1_rv	AAACctgcatcgattctcctgcat
*acbB*_sp1_fw	ACGCggattgtagcaatgccgcca
*acbB*_sp1_rv	AAACtggcggcattgctacaatcc
*acbV*_sp1_fw	ACGCtagatttcgcggtcatcggg
*acbV*_sp1_rv	AAACcccgatgaccgcgaaatcta
*0038*_sp1_fw	AAACtctgtcaagtgcctgttatc
*0038*_sp1_rv	ACGCgataacaggcacttgacaga
*0424*_sp1_fw	AAACtgcggcgcaaggtactctgt
*0424*_sp1_rv	ACGCacagagtaccttgcgccgca
*0500*_sp1_fw	AAACgttccataataggatgatat
*0500*_sp1_rv	ACGCatatcatcctattatggaac
*0946*_sp1_fw	AAACatgaaacacggcgatgaaac
*0946*_sp1_rv	ACGCgtttcatcgccgtgtttcat
*0976*_sp1_fw	AAACatatggtgactccgtggcga
*0976*_sp1_rv	ACGCtcgccacggagtcaccatat
*0989*_sp1_fw	AAACacaaagtggcagcttagaag
*0989*_sp1_rv	ACGCcttctaagctgccactttgt
*1196*_sp1_fw	AAACcgcaacaacttgtacgctac
*1196*_sp1_rv	ACGCgtagcgtacaagttgttgcg
*1252*_sp1_fw	AAACcagtacttcgagctgaccaa
*1252*_sp1_rv	ACGCttggtcagctcgaagtactg
*1572*_sp1_fw	AAACcgaggccggataacagatcc
*1572*_sp1_rv	ACGCggatctgttatccggcctcg
*1607*_sp1_fw	AAACtgtgctaagagtgatcgttc
*1607*_sp1_rv	ACGCgaacgatcactcttagcaca
*1631*_sp1_fw	AAACatctggtctggcacgctgta
*1631*_sp1_rv	ACGCtacagcgtgccagaccagat
*1755*_sp1_fw	AAACtgatctgacaggctgaacag
*1755*_sp1_rv	ACGCctgttcagcctgtcagatca
*1816*_sp1_fw	AAACtatctgaccgcactgtctgc
*1816*_sp1_rv	ACGCgcagacagtgcggtcagata
*1877*_sp1_fw	AAACacggctccacatcgccggcc
*1877*_sp1_rv	ACGCggccggcgatgtggagccgt
*2235*_sp1_fw	AAACacaggtgacacacgcgctgc
*2235*_sp1_rv	ACGCgcagcgcgtgtgtcacctgt
*2344*_sp1_fw	AAACtgttaacttaacagcgttag
*2344*_sp1_rv	ACGCctaacgctgttaagttaaca
*2411*_sp1_fw	AAACtggcatgatttcggtgccgg
*2411*_sp1_rv	ACGCccggcaccgaaatcatgcca
*2872*_sp1_fw	AAACccggatagtagggttcggcc
*2872*_sp1_rv	ACGCggccgaaccctactatccgg

*3384*_sp1_fw	AAACtcccgtaaccggacgcagcg
*3384*_sp1_rv	ACGCcgctgcgtccggttacggga
*3560*_sp1_fw	AAACttaggtaagctcgtggcatg
*3560*_sp1_rv	ACGCatgccacgagcttacctaa
*4202*_sp1_fw	AAACccataatatgtgctcatgca
*4202*_sp1_rv	ACGCtgcatgagcacatattatgg
*4228*_sp1_fw	AAACtgtcatgcgtgtttccgctg
*4228*_sp1_rv	ACGCcagcggaaacacgcatgaca
*4364*_sp1_fw	AAACagcgtcggggcatgacgcct
*4364*_sp1_rv	ACGCaggcgtcatgccccgacgct
*4574*_sp1_fw	AAACacagacgccctgctgcacgc
*4574*_sp1_rv	ACGCgcgtgcagcagggcgtctgt
*4697*_sp1_fw	AAACaggtgcgggacttcctgacg
*4697*_sp1_rv	ACGCcgtcaggaagtcccgcacct
*4864*_sp1_fw	AAACtactctgatcggcatgacag
*4864*_sp1_rv	ACGCctgtcatgccgatcagagta
*4891*_sp1_fw	AAACgtcagtagtttacttgccgc
*4891*_sp1_rv	ACGCgcggcaagtaaactactgac
*5191*_sp1_fw	AAACctccgttaattaacaggggg
*5191*_sp1_rv	ACGCccccctgttaattaacggag
*5298*_sp1_fw	AAACctagaatcactggtccggcg
*5298*_sp1_rv	ACGCcgccggaccagtgattctag
*5377*_sp1_fw	AAACttgttaacggtaacagtttg
*5377*_sp1_rv	ACGCcaaactgttaccgttaacaa
*6401*_sp1_fw	AAACcggtgactcgtgcaggccgg
*6401*_sp1_rv	ACGCccggcctgcacgagtcaccg
*6463*_sp1_fw	AAACgcgctcgcaaactccgtacc
*6463*_sp1_rv	ACGCggtacggagtttgcgagcgc
*6528*_sp1_fw	AAACagatgcagcactggcgcgcg
*6528*_sp1_rv	ACGCcgcgcgccagtgctgcatct
*6859*_sp1_fw	AAACgacctcacggtgatcggcga
*6859*_sp1_rv	ACGCtcgccgatcaccgtgaggtc
*7140*_sp1_fw	AAACcggcacgcggtaacatcgct
*7140*_sp1_rv	ACGCagcgatgttaccgcgtgccg
*7246*_sp1_fw	AAACatatatcgtctaactatcga
*7246*_sp1_rv	ACGCtcgatagttagacgatatat
*7269*_sp1_fw	AAACtcatagttatattgttctgt
*7269*_sp1_rv	ACGCacagaacaatataactatga
*7677*_sp1_fw	AAACggctgctcgaacggtccggg
*7677*_sp1_rv	ACGCcccggaccgttcgagcagcc
*7823*_sp1_fw	AAACcaacattggcactcggagtt
*7823*_sp1_rv	ACGCaactccgagtgccaatgttg
*7951*_sp1_fw	AAACcgtcgtcaacgggtccacca
*7951*_sp1_rv	ACGCtggtggacccgttgacgacg
*7958*_sp1_fw	AAACcaggacatcgggacgttcat
*7958*_sp1_rv	ACGCatgaacgtcccgatgtcctg
*7982*_sp1_fw	AAACcccgtcggtgacgccgggcc
*7982*_sp1_rv	ACGCggcccggcgtcaccgacggg
*8007*_sp1_fw	AAACgacagtgtcgtcatcgccct
*8007*_sp1_rv	ACGCagggcgatgacgacactgtc
*8014*_sp1_fw	AAACaatctcgccgacatcgccga
*8014*_sp1_rv	ACGCtcggcgatgtcggcgagatt
*8048*_sp1_fw	AAACtagacacagcgaaaggggtt

*8048*_sp1_rv	ACGCtagacacagcgaaaggggtt
*8114*_sp1_fw	AAACagactgatcgagcgcagacg
*8114*_sp1_rv	ACGCcgtctgcgctcgatcagtct
*8120*_sp1_fw	AAACctggcgtcctacgtgccggc
*8120*_sp1_rv	ACGCgccggcacgtaggacgccag
*8173*_sp1_fw	AAACaacgtcgtattcttctgcgc
*8173*_sp1_rv	ACGCgcgcagaagaatacgacgtt
*8200*_sp1_fw	AAACctgtatcaggcgaaggccga
*8200*_sp1_rv	ACGCtcggccttcgcctgatacag
*8287*_sp1_fw	AAACctgagcagctcatctcgttc
*8287*_sp1_rv	ACGCgaacgagatgagctgctcag

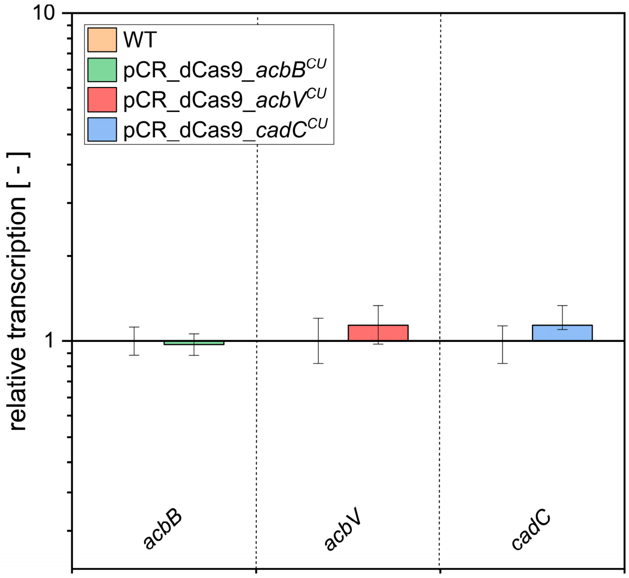

Figure S1. Relative transcription of *acbB*, *acbV*, and *cadC* in the respective, cured strains pCR_dCas9_*acbB*^{CU}, pCR_dCas9_*acbV*^{CU}, and pCR_dCas9_*cadC*^{CU} compared to the wild type (WT). Colors are used to label the different strains. Mean values and standard deviations of three biological replicates are presented. The significance (p-value < 0.05, labeled by *) was determined by Student's t-test (unpaired, two-sided).

Table S4. CRISPRi library target selection criteria according to DNA-affinity chromatography ("regulator fishing", RF), transcriptional dynamics (TD), growth phase (GP), CadC paralogs (CP), and patents (PA). More than one criterion may apply. Measured data of the library are shown with CDW and acarbose concentration from three biological replicates.

target	selection criteria	CDW [g L^{-1}]	acarbose concentration[g L^{-1}]
ACSP50_*0038*	RF	13.467 ± 0.40	0.501 ± 0.014
ACSP50_*0424*	TD	13.167 ± 1.15	0.430 ± 0.002
ACSP50_*0500*	RF	14.900 ± 0.52	0.622 ± 0.026
ACSP50_*0946*	RF	13.800 ± 1.31	0.454 ± 0.003
ACSP50_*0976*	RF	11.500 ± 0.69	0.407 ± 0.004
ACSP50_*0989*	RF	11.900 ± 0.89	0.389 ± 0.001
ACSP50_*1196*	RF	13.633 ± 1.72	0.496 ± 0.010
ACSP50_*1252*	RF	12.400 ± 1.04	0.395 ± 0.011
ACSP50_*1572*	RF	12.467 ± 1.12	0.400 ± 0.005
ACSP50_*1607*	RF	13.633 ± 1.21	0.416 ± 0.008
ACSP50_*1631*	RF; TD	12.767 ± 0.40	0.450 ± 0.002
ACSP50_*1755*	CP	11.600 ± 0.75	0.415 ± 0.004

ACSP50_*1816*	RF, PA	14.400 ± 0.70	0.474 ± 0.002
ACSP50_*1877*	TD	12.100 ± 0.62	0.577 ± 0.005
ACSP50_*2235*	TD	13.133 ± 1.96	0.443 ± 0.005
ACSP50_*2344*	TD	12.967 ± 1.26	0.390 ± 0.002
ACSP50_*2411*	TD	13.567 ± 1.12	0.531 ± 0.002
ACSP50_*2872*	RF	13.100 ± 0.53	0.429 ± 0.013
ACSP50_*3384*	RF	15.033 ± 0.76	0.540 ± 0.027
ACSP50_*3560*	RF	12.967 ± 1.78	0.448 ± 0.002
ACSP50_*4202*	RF, CP	13.633 ± 1.16	0.619 ± 0.021
ACSP50_*4228*	RF	14.200 ± 0.70	0.391 ± 0.002
ACSP50_*4364*	TD	11.600 ± 1.00	0.349 ± 0.001
ACSP50_*4574*	CP	15.833 ± 0.31	0.472 ± 0.003
ACSP50_*4697*	TD	15.367 ± 0.90	0.558 ± 0.004
ACSP50_*4864*	RF	16.000 ± 2.57	0.524 ± 0.002
ACSP50_*4891*	RF	13.300 ± 2.52	0.501 ± 0.010
ACSP50_*5191*	CP	17.667 ± 1.01	0.345 ± 0.017
ACSP50_*5298*	RF	13.833 ± 1.42	0.460 ± 0.012
ACSP50_*5377*	RF, PA	12.800 ± 1.73	0.430 ± 0.008
ACSP50_*6401*	TD	15.533 ± 1.66	0.610 ± 0.005
ACSP50_*6463*	RF, TD, PA	13.700 ± 0.75	0.062 ± 0.007
ACSP50_*6528*	RF	12.567 ± 0.75	0.353 ± 0.018
ACSP50_*6859*	RF	13.700 ± 0.75	0.521 ± 0.003
ACSP50_*7140*	RF	13.333 ± 0.96	0.571 ± 0.001
ACSP50_*7246*	RF	12.800 ± 1.39	0.431 ± 0.012
ACSP50_*7269*	RF	13.867 ± 1.47	0.594 ± 0.029
ACSP50_*7677*	RF	14.733 ± 0.47	0.546 ± 0.010
ACSP50_*7823*	RF	11.733 ± 0.38	0.567 ± 0.004
ACSP50_*7951*	RF	11.933 ± 0.83	0.394 ± 0.003
ACSP50_*7958*	RF	11.500 ± 0.90	0.367 ± 0.003
ACSP50_*7982*	RF	11.633 ± 0.35	0.461 ± 0.002
ACSP50_*8007*	TD	14.233 ± 1.45	0.585 ± 0.013
ACSP50_*8014*	RF	13.067 ± 0.93	0.501 ± 0.016
ACSP50_*8048*	RF	13.267 ± 0.15	0.415 ± 0.003
ACSP50_*8114*	RF	13.200 ± 0.40	0.498 ± 0.002
ACSP50_*8120*	TD	12.267 ± 1.07	0.341 ± 0.016
ACSP50_*8173*	RF	11.200 ± 0.46	0.296 ± 0.009
ACSP50_*8200*	TD	12.933 ± 1.81	0.469 ± 0.031
ACSP50_*8287*	TD	11.733 ± 1.01	0.459 ± 0.005
